# Editorial on the Special Issue on Microelectrode Arrays and Application to Medical Devices

**DOI:** 10.3390/mi11080776

**Published:** 2020-08-14

**Authors:** Alinaghi Salari, Colin Dalton

**Affiliations:** 1 Institute for Biomedical Engineering, Science and Technology (iBEST), Toronto, ON M5B 1T8, Canada; a1salari@ryerson.ca; 2 Electrical and Computer Engineering Department, University of Calgary, Calgary, AB T2N 1N4, Canada

In this editorial note, we briefly review the major findings of the 10 articles published in the Special Issue on microelectrode arrays and application to medical devices. The articles are categorized into three sections, i.e., fabrication techniques [1,2,3,4], application demonstration [5,6,7,8], and review of recent advances in this field [9,10].

## 1. Fabrication Techniques

In many drug screening and electrophysiological applications, microelectrodes are commonly used to study electric field and impedimetric measurements of cells in vitro. In order to couple such a platform with optical microscopy, indium tin oxide (ITO), which is a transparent material, can be used instead of titanium nitride (TiN). The deposition of ITO and TiN thin films are commonly conducted by the sputter deposition technique. However, Ryynänen et al. successfully demonstrated that ion beam-assisted electron deposition (IBAD) can be used as an alternative method for this purpose [1]. They studied three different approaches for the fabrication of microelectrodes. First, an optimal imaging capability was demonstrated when the tracks and electrodes were both made of ITO. In the second approach, a thin layer of TiN was coated onto the electrodes in order to decrease the electrical impedance. To obtain the optimal electrical performance, in the third approach, the electrodes were made of opaque TiN electrodes. In their study, the electrical impedance and noise levels, light transmission through the substrate, and biocompatibility of the fabricated devices were also analyzed.

Laser micromachining is another technique used for electrode microfabrication, which involves the ablation of materials, and are available in a wide range of wavelengths, pulse durations, and repetition rates. When operated in the femtosecond regime, this technique can be useful for the fabrication of sub-10-micron features. However, this operational regime can be costly and suffers from high power consumption. Hart et al. characterized a multimodal laser micromachining tool featuring limited power consumption and nanosecond ablation [2]. They showed that these features provide better control over the material selectivity for laser micromachining and ablation depths. They also analyzed the ablation characteristics of six different materials and demonstrated the application of this tool for the laser micromachining of shadow masks with a 1.5-µm feature size and microelectrodes with 7-µm electrode gas widths.

For the application of implantable microelectrode arrays, the material and biocompatibility of such devices are crucial. Intracortical microelectrode arrays, for example, can be used for recording electrical function or neural stimulation in brain tissue. Due to the disadvantages of the application of stiff and brittle materials for the fabrication of such implants, polymer materials, which enable robust, yet softer, properties have been suggested. Still, a successful implant of a polymer-based device in brain tissue can be challenging and, as a result, further fabrication steps including temporary stiffening coatings or insertion guides are often required. To overcome these limitations, Stiller et al. have investigated the use of shape memory polymers (SMP) in intracortical devices [3]. They fabricated an intracortical recording probe using a thiol-ene/acrylate formulation, which features a dynamic response to stimuli from the environment, meaning that the modulus of elasticity of such material changes considerably (by two orders of magnitude) when its environment is changed from room temperature and dry conditions to body temperature and wet conditions. The authors also conducted performance characterization and demonstrated in vivo intracortical recordings over several weeks in a rat brain, suggesting that this SMP device is a reliable candidate for such applications.

In another study, Wang et al. investigated the fabrication of a microelectrode array in the form of pyramidal microstructures on a polydimethylsiloxane (PDMS) substrate with a parylene transition layer between the electrode and the substrate [4]. Their results showed that this method of fabrication can increase the contact area of the electrodes, enhance the bonding force between the substrate and the electrodes, and reduce the electrode impedance as well as the electrode–skin contact impedance. This fabrication method can help the development of wearable and implantable medical devices.

## 2. Application Demonstration

Monitoring the local cerebral tissue oxygen levels (PbtO_2_) can provide critical information about brain function including neurovascular metabolic activity. Different invasive and non-invasive techniques have been reported for measuring the tissue oxygen levels. Among those, the amperometric sensing technique allows real-time monitoring of brain oxygenation and can be an effective bedside method for the detection of cerebral ischemia. Due to the electrocatalytic behavior of Pt, Ledo et al. reported the potential application of those commercially available intracranial recording electrodes that include a thin Pt film on their recording sites, for amperometric monitoring of PbtO_2_ [5]. They conducted a surface morphology characterization and an electrochemical evaluation of the performance of such a device for oxygen detection and concluded that these probes can be repurposed for in vivo multisite monitoring of PbtO_2_.

The study of cellular electrophysiology in in vitro settings can be performed using microelectrode arrays, which can conduct extracellular recordings of various chemicals, such as glucose. This application was demonstrated by Alassaf et al., who studied the function of human islets by measuring the electrical activities of dissociated islet cells in real-time and over prolonged culture periods using microelectrode arrays [6]. Compared with glucose-stimulated insulin secretion assays, their results showed a correlation between the electrical activities and the functional secretory response of the islet cells. Their measurements using a microelectrode array platform exhibited a highly sensitive and rapid assessment of islet functionality, demonstrating its potential application for studying islet physiology.

Another important application of microelectrodes is in biosensing technology. Bisphenol A (BPA) is an organic chemical which can be detrimental to various organ systems including the endocrine, reproductive, and nervous systems. In an effort to enhance the detection capabilities of BPA in serum samples, Luo et al. coupled self-assembly technology with AC electrokinetics (ACEK) and showed that this strategy could yield a rapid and sensitive detection of BPA antigens in fluid samples [7]. In their work, the surfaces of microelectrodes were functionalized with the BPA antibody via self-assembly, while an AC electric field was used to actuate the electrodes. Compared to diffusion-limited sensing approaches, the ACEK microflows enhanced the transport of antigens and, thus, improved the antigen-antibody binding. The interfacial capacitance was then measured in order to analyze the concentration of the bound antigens. This process resulted in the successful detection of nanomolar levels of BPA within one minute of operation, signifying its potential application in the detection of biochemical molecules.

In another study on electrokinetics, the transient motion of Janus particles acting as mobile microelectrodes was studied by Liu et al. [8]. A Janus particle is a type of particle that has a nonuniform polarizable body, such that half of its body is less polarizable than the surrounding liquid, while the other half is more polarizable. With this special configuration, particle translation due to nonlinear induced-charge electrophoresis (ICEP) can occur even in the presence of a uniform electric field. In this work, the authors numerically investigated the motion of cylindrical Janus polystyrene particles with half bodies covered with thin films of a polarizable metal (e.g., gold). In their 2D simulation, the transient electrokinetic behavior of these particles was studied by considering the induced-charge electroosmotic flow and Maxwell–Wager interfacial polarization, assuming that the particles were freely suspended in an electrolyte far from the channel walls. They reported a new electrokinetic transport phenomenon, called ego-dielectrophoresis, which is dominant at high frequency ranges (e.g., 100 MHz). They also showed that this new electrokinetic phenomenon causes the direction of particle motion to reverse as the actuation frequency transitions from low (e.g., 100 Hz) to high (e.g., 100 MHz) ranges. This AC electrokinetics of Janus microelectrodes can be particularly useful for the detection of biomolecules in lab-on-a-chip devices.

## 3. Review of Recent Advances in the Field

As an emerging technology, wearable and flexible sensing have been widely studied due to their important role in personalized medicine and their capabilities in real-time monitoring of an individual’s health. The application of liquid metals, which can provide a higher degree of flexibility and conductivity compared to conventional soft sensors, in different biomedical areas was reviewed by Ren et al. [9]. The authors presented the recent progress of liquid metal-enabled soft sensors from different aspects, including innovations in material selection and fabrication techniques, fundamental principles, and typical applications. The existing challenges and possible development directions in this area were also pointed out in their article.

As discussed earlier, the AC electrokinetic effect can be useful for sample enrichment and fluid and particle transport in microfluidic devices. In a review article, Salari et al. discussed recent advances in this field with a focus on AC electrothermal techniques from different aspects involving external electric field configurations, temperature field, and the resultant velocity field [10]. The equations governing electrothermal effect were first presented, and then various strategies for the generation of electrothermal microflows were reviewed. The major applications of such flows in microfluidics and key points in numerical simulations and experimental AC electrothermal devices were covered. The authors also discussed some current limitations and future directions in this research field.

At the end of this editorial note, we would like to thank all the authors who contributed to this Special Issue and also to acknowledge the time and effort dedicated by the reviewers to improve the quality of the published articles.
